# Supplementary material for: Selective translation of epigenetic modifiers affects the temporal pattern and differentiation of neural stem cells
Source: Nat Commun. 2022 Jan 25;13:470. doi: 10.1038/s41467-022-28097-y (PMC8789897; doi:10.1038/s41467-022-28097-y)
Supplement: Supplementary file 1 — Supplementary Information [file 41467_2022_28097_MOESM1_ESM.pdf]

Supplymentary Information file

Selective translation of epigenetic modifiers affects the temporal pattern and differentiation of neural stem cells

Quan Wu , Yuichi Shichino , Takaya Abe , Taeko Suetsugu , Ayaka Omori , Hiroshi Kiyonari , Shintaro Iwasaki , Fumio Matsuzaki

Supplymentary Figure 1-10

Supplymentary Fig.1

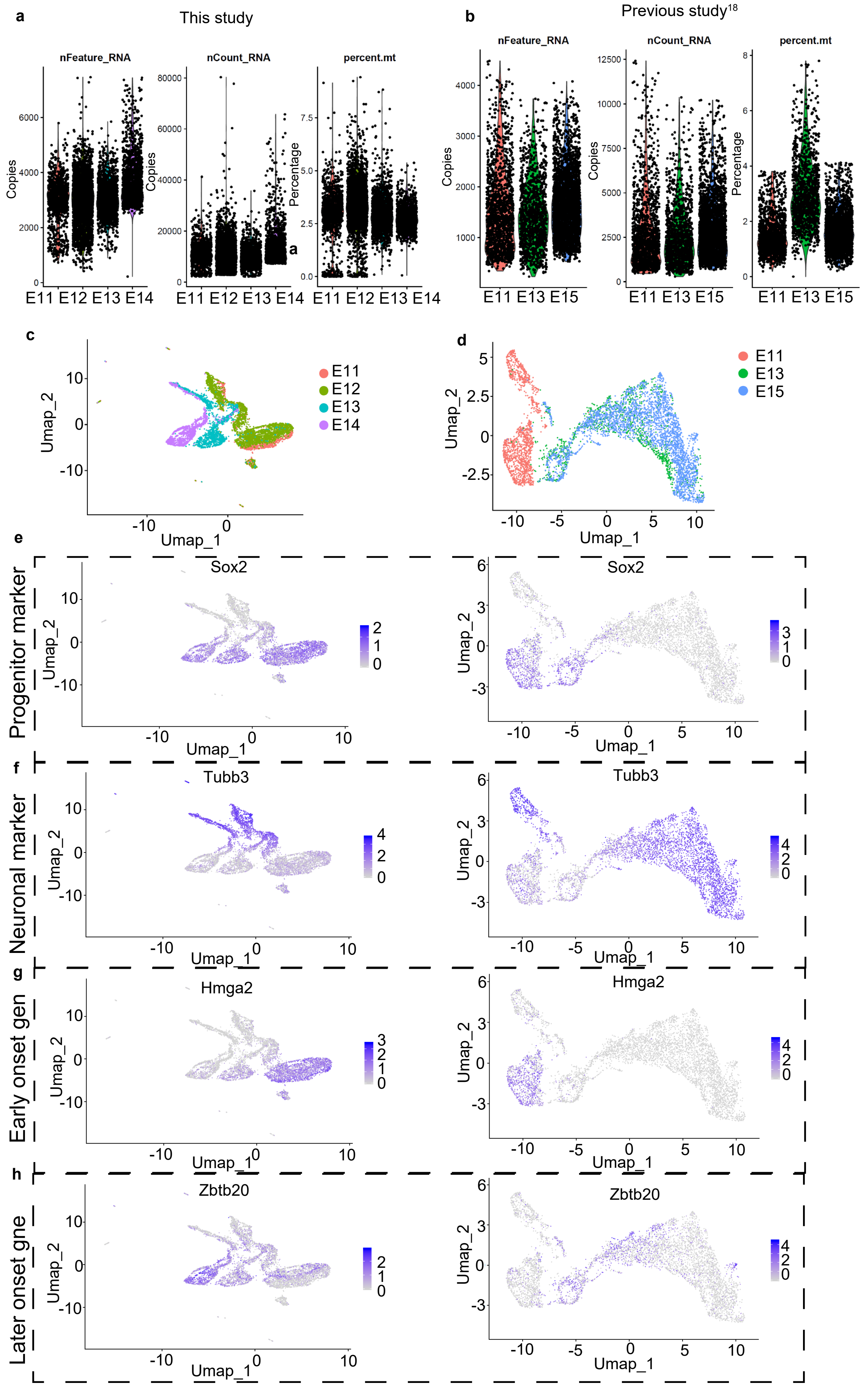

Supplementary Fig.1 Comparisons of the reuslts of single cell RNA-seq in our study with the previous study. a,b In our study (a) and in a preivous study[18] (b), the number of detected genes (nFeature), unique molecular index (nCount) and percentage of mitochondrial genes in each cell was plotted. **c,d** Scatterplot of the single-cell transcriptome after uniform manifold approximation and projection (UMAP), coloured by genotypes from different stages. **e-h** Comparisons of the expression pattern of serveal markers in two studies.

Supplymentary Fig2

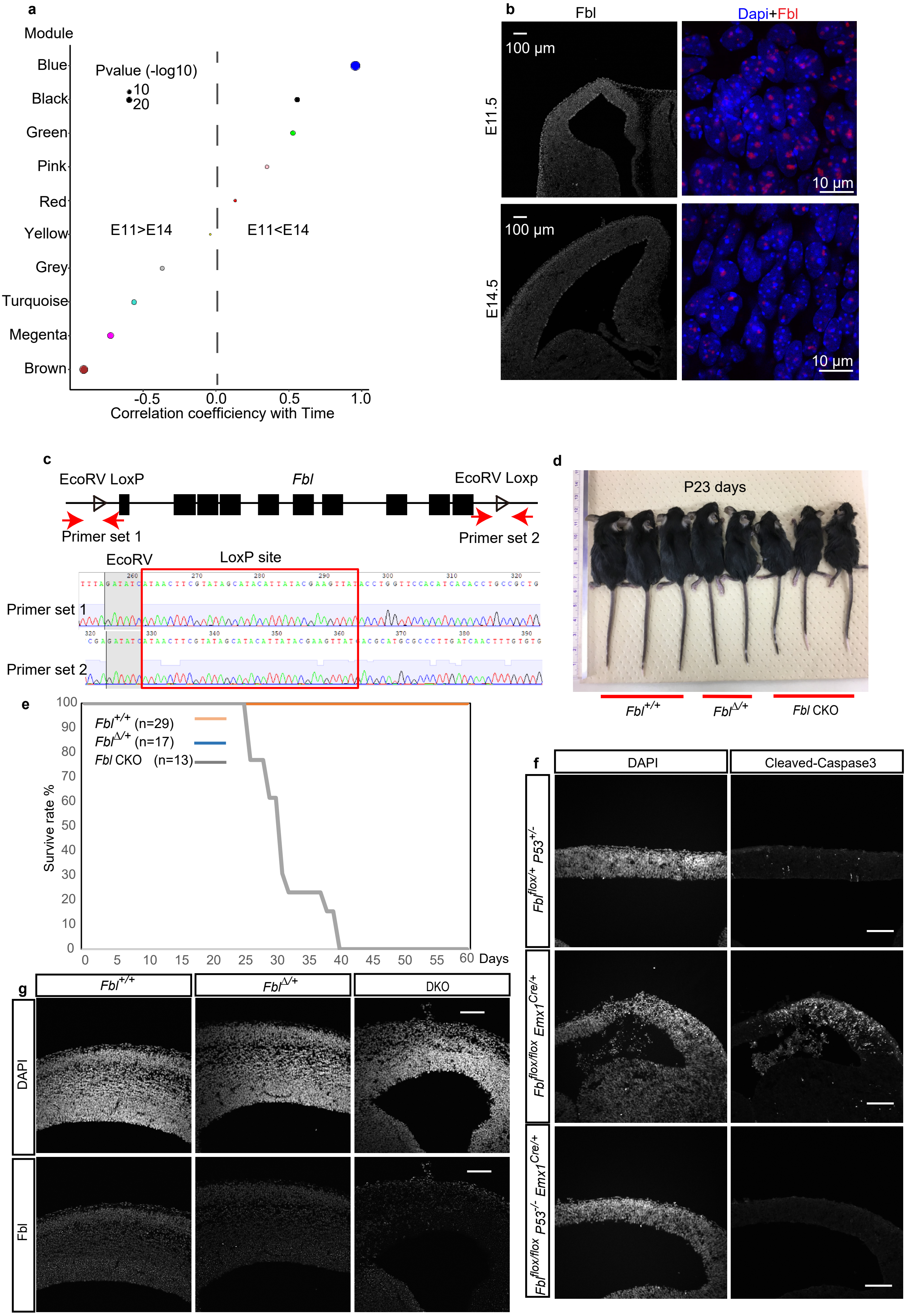

Supplementary Fig. 2 Identification of Fbl as a condidate to promote the developemental clock in neural stem cells (NSCs). a WGCNA gene dendrogram classifies E11 and E14 NSCs into different modules and correlation coefficient of each module with time (E11 and E14). If the correlation coefficient of the module is closer to -1 and 1, genes in that module are highly expressed in E11 and E14 NSCs, respectively. The colored dots represents different modules. **b** Representative image of E11 and E14 brain sections stained for Fbl. Scale bar: 10 or 100 μm. **c** Schematics of the CRISPR-CAS9-dependent knock-in of loxP sites flanking Fbl. Confirmation of loxP sites by sequencing analysis. **d** Appearance of $Fbl^{+/+}$, $Fbl^{\Delta/+}$, and Fbl CKO pups at 23 days postnatal. **e** Survival rate of $Fbl^{+/+}$, $Fbl^{\Delta/+}$, and Fbl CKO pups. **f** Representative image of E12 brain section stained for cleaved-caspase 3 antibody. Scale bar: 100 μm. **g** Representative image of the E14 brain section stained for Fbl. Scale bar: 100 μm.

Supplymentary Fig.3

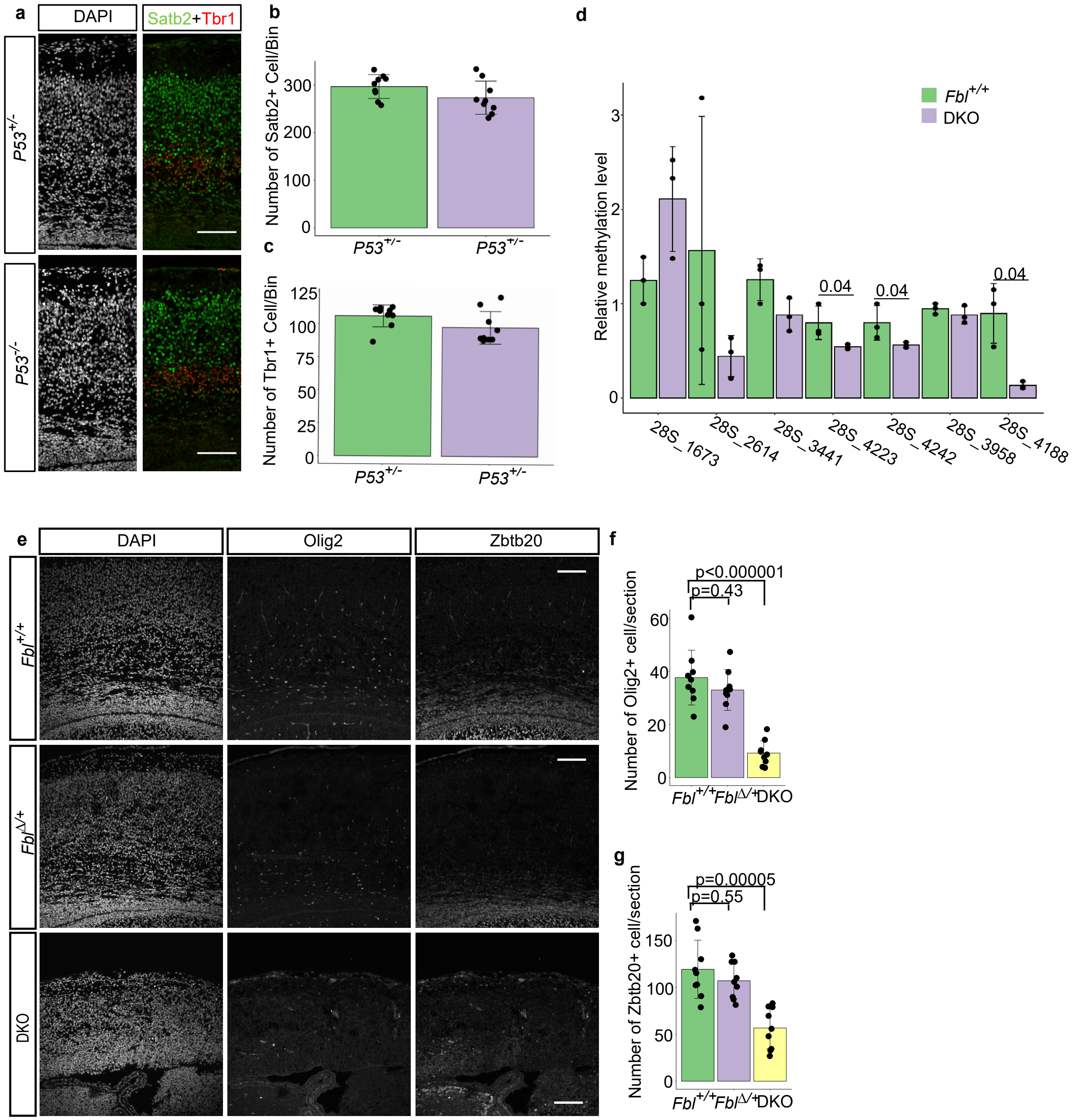

Supplementary Fig. 3 Deletion of *Trp53* does not affect neurogenesis, while *Fbl* knockout affects methylation of rRNA, oligogenesis and astrocytogenesis. a Representative image of *Trp53*$^{+/+}$ and *Trp53*$^{-/-}$ brain sections stained for Satb2 and Tbr1. **b**,**c** Quantification of cell number quantification on sections based on immunostaining with the indicated markers (n=3 mice per genotype, n=3 sections per mouse; data are presented as mean±s.d. of n=9 sections). **d** Methylation level at the indicated sites in *Fbl*$^{+/+}$ and DKO neural stem cells (one-sided Wilcoxon signed rank test; data are presented as mean±s.d of n=3 biologically indepedent aminals). **e** Representative image of E17 brain section stained for Oligo2 and Zbtb20. **f**,**g** Quantification of cell number on sections based on immunostaining with the indicated markers (n=3 mice per genotype, n=3 sections per mouse; one-way ANOVA followed by Tukey's post-hoc tests; data are presented as mean±s.d. of n=9 sections). Scale bar: 100 µm.

Supplymentary Fig.4

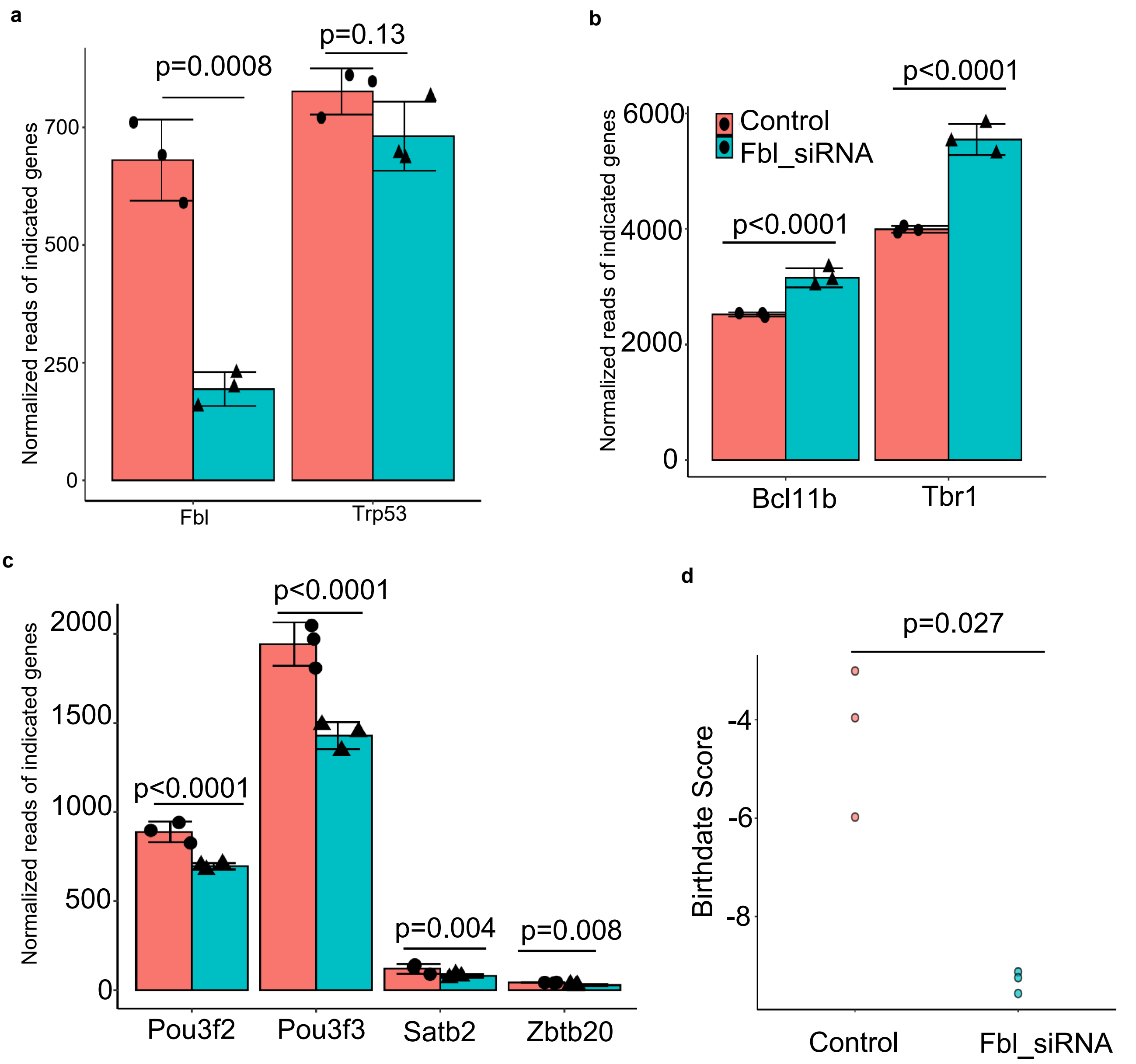

Supplementary Fig. 4 Knockdown of *Fbl* by siRNA influences temporal fate transition of neural stem cells. **a** *Fbl* siRNA reduce the expression of *Fbl* but did not induce the expression *Trp53*. **b,c** Change in expression of deep layer neuron markers (c) and upper layer neuron markers (d). **d** Birthdate score after *Fbl* siRNA treatment for 3 days from E11.5. Two-sided student t-test; data are presented as mean±s.d of n=3 biologically independent samples.

Supplymentary Fig.5

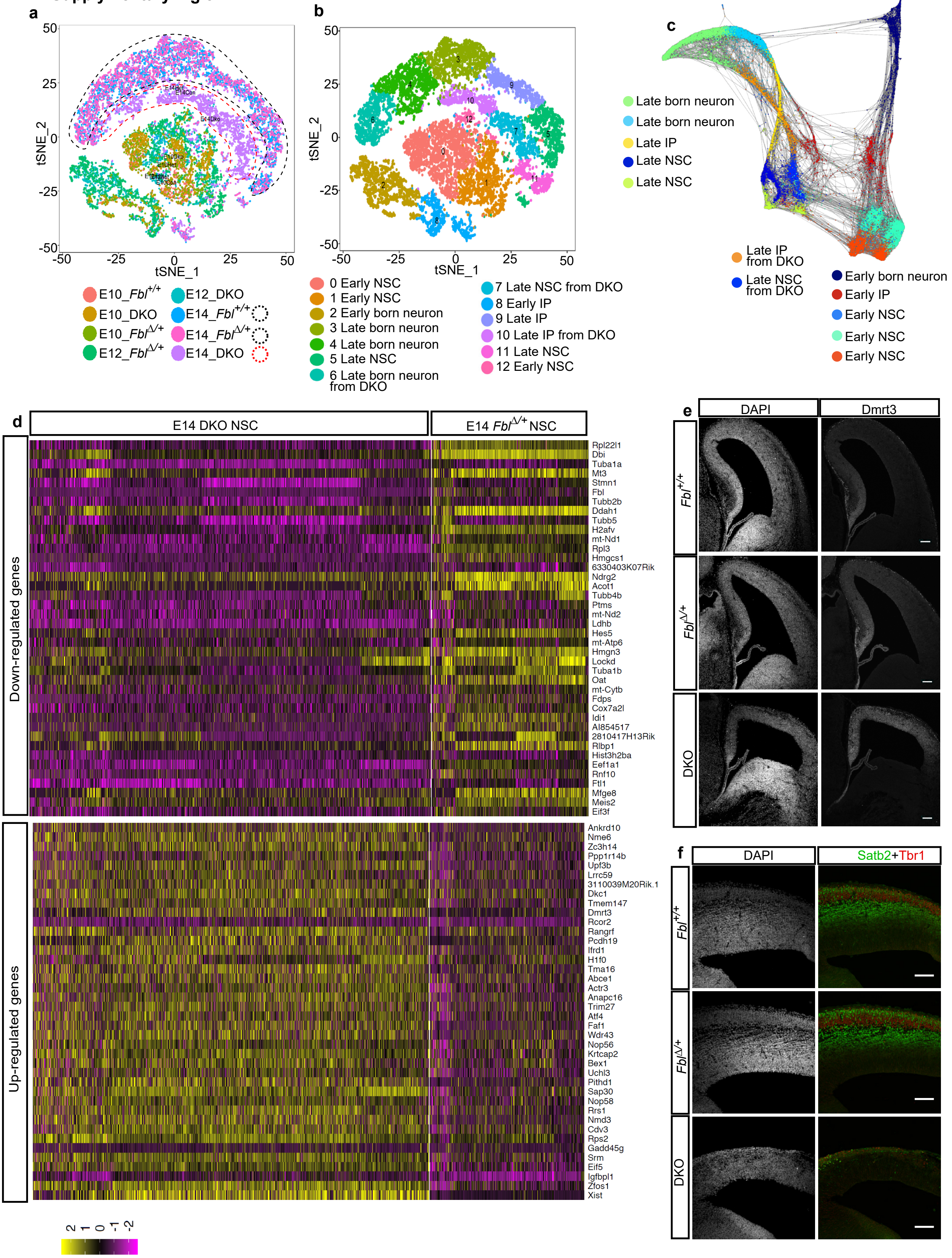

Supplementary Fig. 5 Single-cell transcriptome analysis of temporal pattern in neural stem cells. **a**,**b** Scatterplot of single-cell transcriptome after t-stochastic neighbour embedding (t-SNE), coloured by genotypes from different stages (a) or different cell types (b). In (a), the separation of E14 DKO brain cells from E14 $Fbl^{+/+}$ or $Fbl^{\Delta/+}$ brain cells is highlighted by the dotted red and black circles, respectively. **c** SPRING graph of single cells coloured by cell type identified by the expression of marker genes. **d** Heatmap of the top 40 differentially expressed genes in the comparison of E14 DKO and $Fbl^{\Delta/+}$ NSCs, ranked by fold change. **e**,**f** Representative images of E14 brain sections stained for Dmrt3 (e) and Satb2/Tbr1 (f). Staining was performed in three different biological samples for each genotype. Scale bar: 100 μm.

Supplymentary Fig.6

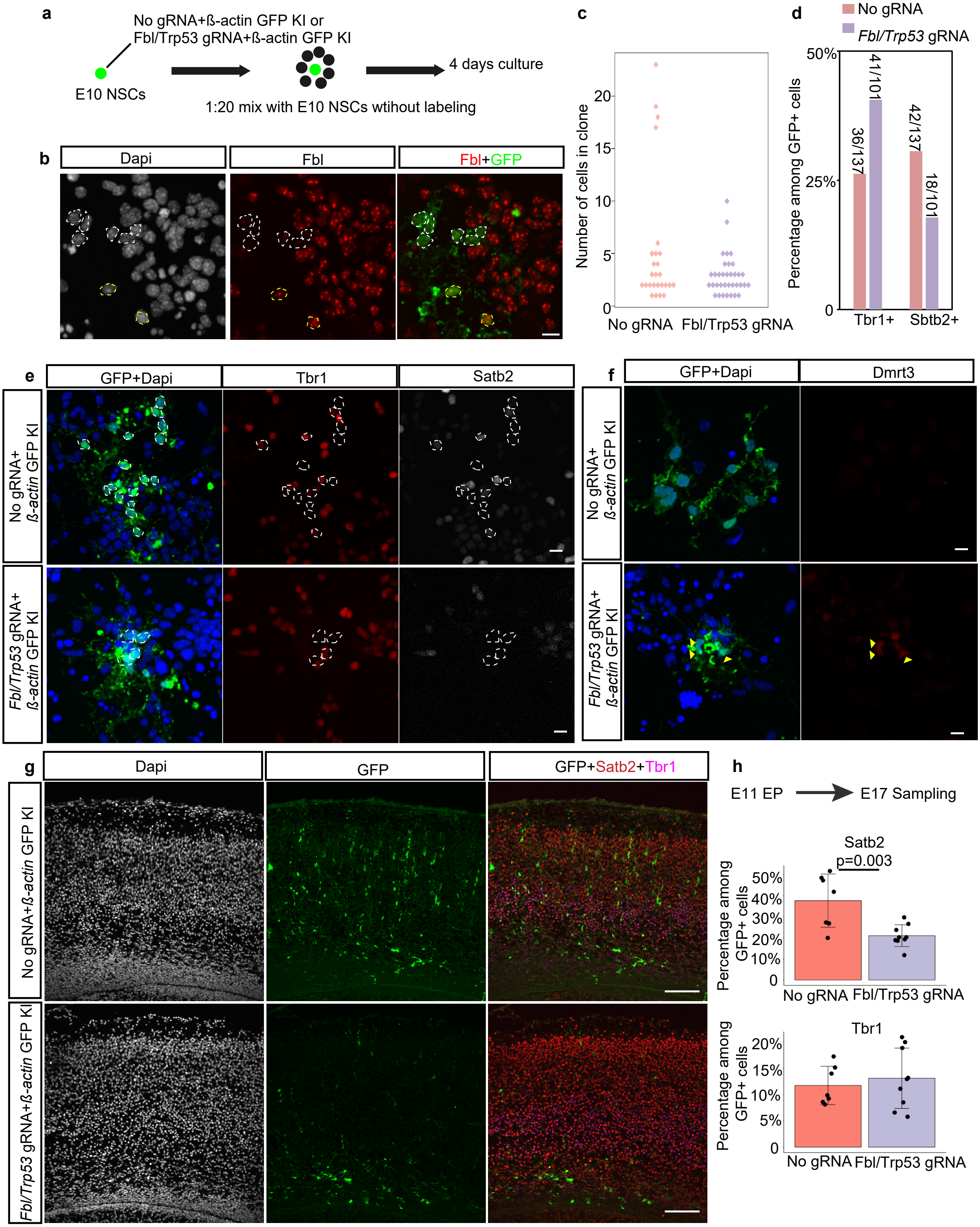

Supplementary Fig.6 Fbl works intrinsically for temporal pattern transition. a Schematics of the experimental design for clonal analysis of *Fbl/Trp53*-null neural stem cells (NSCs). Cells from dorsal brains were electroporated at E10 with/without *Fbl* and *Trp53* gRNA for deletion and with β-actin gRNA for labelling. **b** Representative image of cultured cells stained for Fbl showing deletion of Fbl in some GFP-positive cells (n=39/88). White and yellow circles indicate Fbl-negative and positive cells, respectively. Scale bar: 10 µm **c** Clone size analysis of normal and *Fbl/Trp53*-null NSCs after 4 days of culture. (Clone number n=26 and n=35 for control and knockout, respectively, from two independent experiments). **d** Percentage of Tbr1- and Satb2-positive cells among GFP-positives based on staining. The number of counted cells is shown. **e** Representative image of cultured cells stained for GFP/Dapi, Tbr1, and Brn2. Scale bar: 10 µm. **f** Representative image of cultured cells stained for GFP/Dapi and Dmrt3, showing more *Fbl/Trp53*-null cells (11/39) expressing Dmrt3 than control cells (2/34), which was also observed in *Fbl* DKO brains in Figure S5E. Scale bar: 10 µm. **g** Representative image of E17 brain section stained for GFP, Tbr1, and Satb2. Dorsal brains were electroporated at E11 with/without *Fbl* and *Trp53* gRNA for deletion and β-actin gRNA for labelling. Scale bar: 100 µm. **h** Quantification of Satb2- and Tbr1-positive cells on sections based on immunostaining (n=3 mice per genotype, n=2-3 sections per mouse; Two-sided student's t-test; data are presented as mean±s.d. of counted sections).

Supplymentary Fig.7

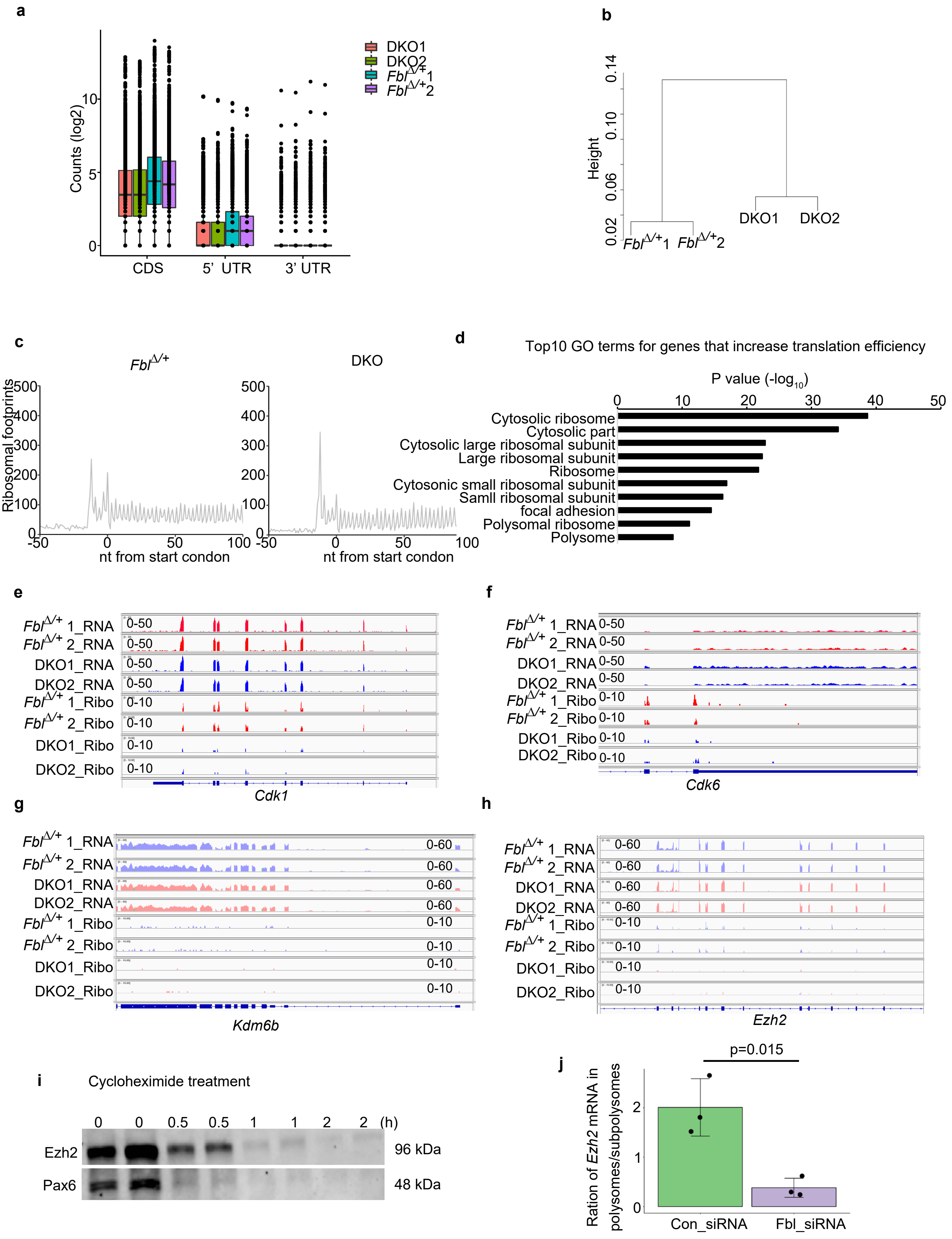

Supplementary Fig. 7 Assessment of translational efficiency (TE) dependent on Fbl via ribosome profiling. **a** Counts of ribosome footprints mapping on different mRNA regions (n=2 of biologically independent samples). Data in the box-whiskers show maximum (except outliers), third quartile to first quartile, median and minimum (except outliers). Outliers data are plotted as points outside of the box. Outliers are defined as 1.5 folds larger or smaller than interquartile range from third quartile or first quartile, respectively. **b** Hierarchical clustering of ribosome profiling data. **c** Metagene analysis of the 5' end of footprints showing three-nucleotide periodicity of the ribosomal footprint reads. **d** Top 10 GO terms of transcripts whose TE increased after *Fbl* knockout. **e-h** Genome browser view of RNA-seq and Ribo-seq density of the indicated genes. **i** Western bloting analysis of Ezh2 and Pax6 after treatment with 100 µg/ml cycloheximide for the indicated time, showing that Pax6 was not more stable than Ezh2 (n=2). **j** The ratio of the expression of *Ezh2* mRNA level in subpolysome and polysome after treatment with control and *Fbl* siRNA (Data are presented as the mean±s.d.of n=3 independent experiments). P-value is calculated by Student's t-test (two-sided).

Extended Data Fig.8

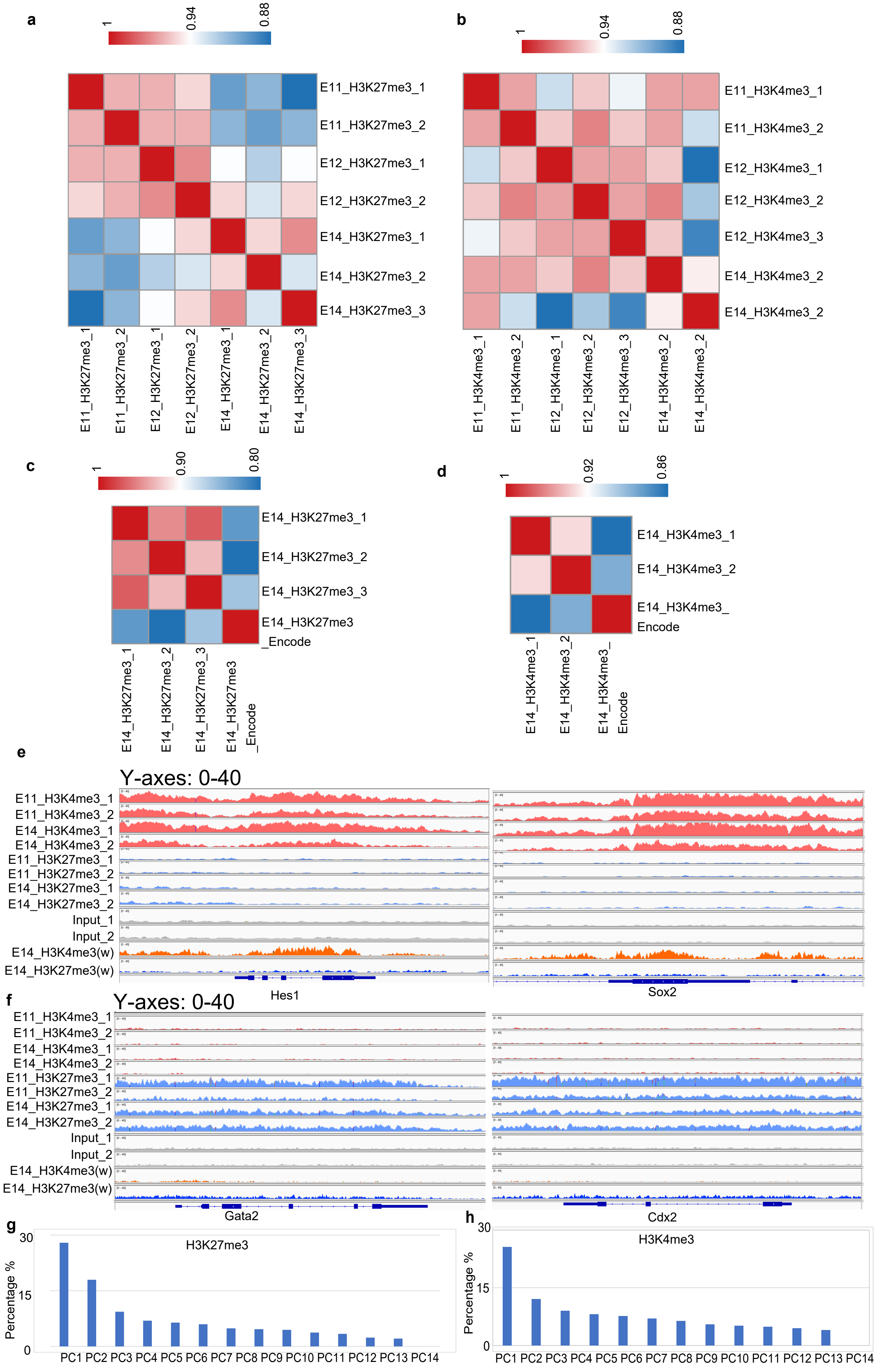

Supplementary Fig. 8 Quality check of single cell RNA-seq and ChIP-seq experiments. a SPRING graphs indicating the expression pattern of a NSC marker: *Pax6*, a deep layer marker: *Tbr1* and an upper layer marker: *Satb2*, respectively. **b**,**c** Sample correlation of ChIP-seq experiments using H3K27me3 (b) and H3K4me3 antibodies (c among E11, E12, and E14 neural stem cells (NSCs). **d**,**e** Sample correlation of ChIP-seq experiments using H3K27me3 (d) and H3K4me3 antibodies (e) between our data from E14 NSCs and published data from E14 whole brains. **f** Genome browser view of ChIP-seq density of the indicated genes. **g**,**h** Contribution of each PC in the PCA of H3K27me3 (g) and H3K4me3 (h).

Supplymentary Fig.9

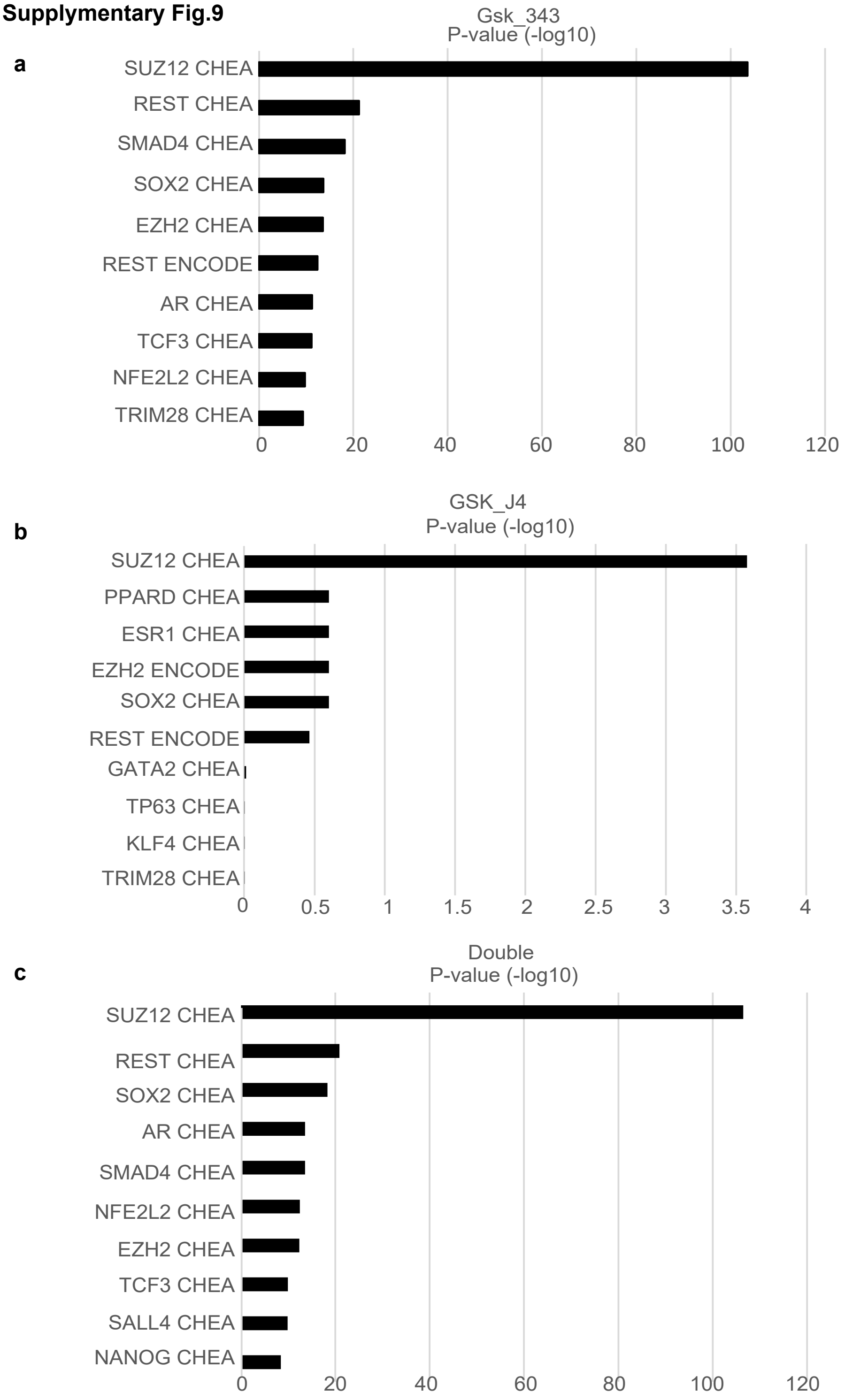

Supplementary Fig. 9 ChIP enrichment analysis using genes that showed signifcant expression changes after treatment with Gsk_343 (a), Gsk_j4 (b) or both (c).

Supplymentary Fig.10

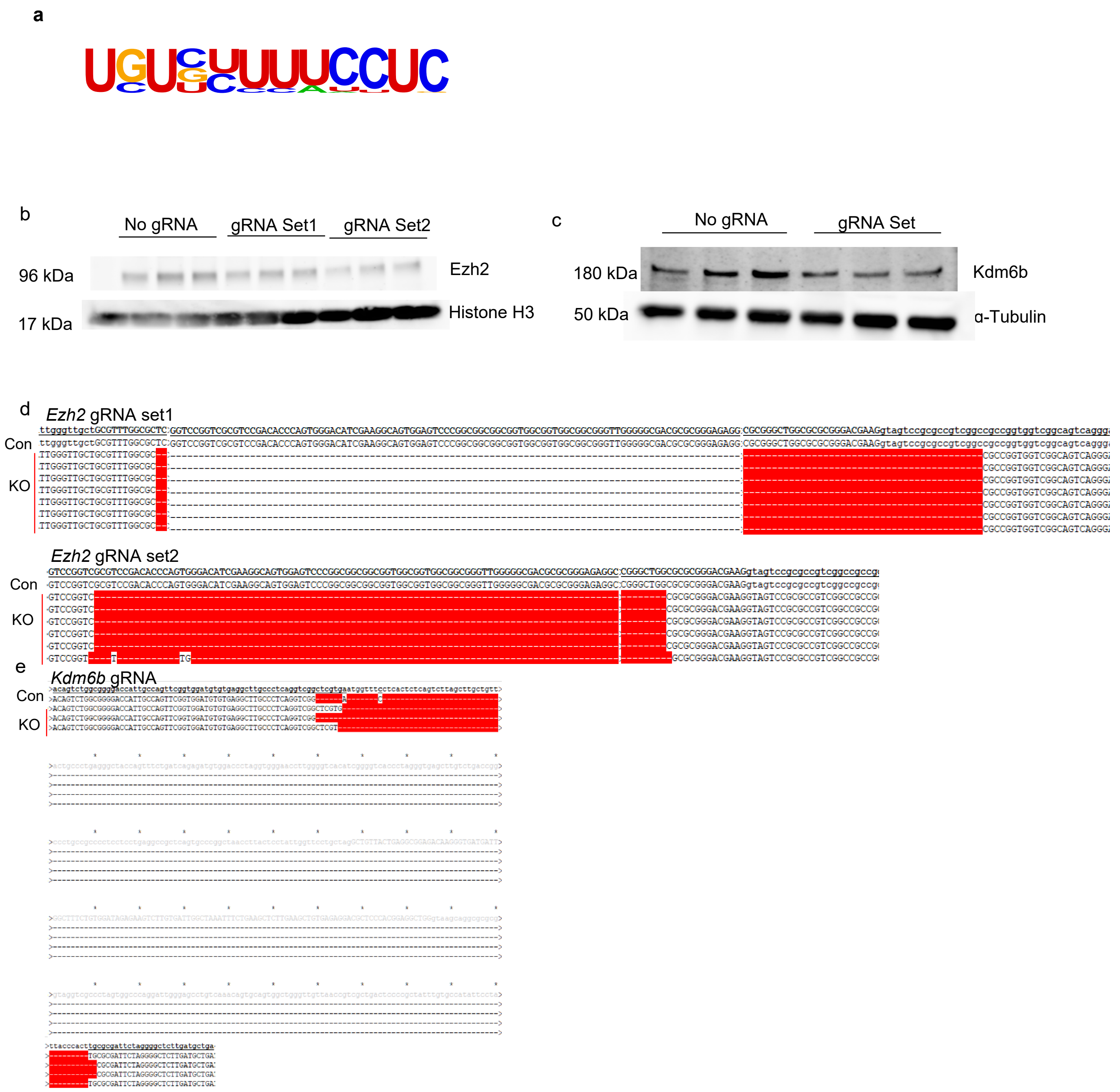

Supplymentary Fig. 10 5′UTR of Ezh2 and Kdmb6 is important for translation of these genes. **a** A poly(U) motif enriched in the 5′UTRs of mRNAs with downregulated translation efficiency. **b,c** Detection of Ezh2 and Kdm6b protein by western bloting after knockout of the 5′UTR of these genes.. **d,e** Confirmation of the deletion of the 5′UTR of Ezh2 (d) and Kdm6b (e) by sequencing.
