# Supplementary material for: Selective translation of epigenetic modifiers affects the temporal pattern and differentiation of neural stem cells
Source: Nat Commun. 2022 Jan 25;13:470. doi: 10.1038/s41467-022-28097-y (PMC8789897; doi:10.1038/s41467-022-28097-y)
Supplement: Supplementary file 3 — Description of additional Supplementary Files [file 41467_2022_28097_MOESM3_ESM.pdf]

DESCRIPTION OF ADDITIONAL SUPPLEMENTARY DATA FILES

Supplementary Data 1: Identification of module genes by WGCNA (a) and the gene list of the brown module (b). (c) Gene list of GO terms shown in Figure 1d.

Supplementary Data 2: a, Gene expression change after knockdown of Fbl using siRNA. The gene expression level in each sample was fitted with negative binormial model and p-value was calculate by likelihood-ratio test. b, Top10 marker genes for identification of each cell type. c-e, DEG between Fbl+/+ and DKO NSCs at E10, E12 and E14, respectively.P-value was calculated by wilcoxon rank sum test. f, PC contribution values of E14 differentially expressed genes (DEG) compared with Fbl+/+ and DKO NSCs.

Supplementary Data 3: The list of genes that downregulated and upregulated their translational efficiency after knockout of Fbl (a) and results of GO analysis for downregulated genes (b) and upregulated genes (c) were also showed. RNA-Seq and ribosome profiling measurements were fitted with generalized linear models, separately. P-value was calculated by likelihood-ratio test.

Supplementary Data 4 : List of genomic regions that changed their intensity of H3K27me3 (a) and H3K4me3 (b) peaks between E11 and E14 NSCs. P-value was calculated by a two-sided Wilcoxon „Mann-Whitney‟ test.

Supplementary Data 5: List of genes that associated with genomic regions that showed higher intensity of H3K27me3 peaks at E11 (a) and E14 (b). The top 10 GO term for these genes was shown in c and d. e, List of genes that associated with genomic regions that showed higher intensity of H3K4me3 peaks at E11 and E14. P-value computed using the Fisher exact test.

Supplementary Data 6: a, List of genomic regions that changed their intensity of H3K27me3 peaks between between E14 FblΔ/+ and DKO brains. P-value was calculated by a two-sided Wilcoxon „MannWhitney‟ test. b-d Gene expression change after treatment of Gsk 343 , Gsk J4 and both inhibitors.The gene expression level in each sample was fitted with negative binormial model and p-value was calculated by likelihood-ratio test. e Expression change of genes, which showed specific H3K27me3 peaks in E14NSCs, comparing E14 Control and DKO NSCs. P-value was calculated by wilcoxon rank sum test.

Supplementary Data 7: Sequencing and mapping information for scRNA (a), ChIP-seq (b) and RNA-seq (c) used in this study.
