# Supplementary material for: Selective translation of epigenetic modifiers affects the temporal pattern and differentiation of neural stem cells
Source: Nat Commun. 2022 Jan 25;13:470. doi: 10.1038/s41467-022-28097-y (PMC8789897; doi:10.1038/s41467-022-28097-y)
Supplement: Supplementary file 5 — Reporting Summary [file 41467_2022_28097_MOESM5_ESM.pdf]

nature research

Corresponding author(s): Fumio Matsuzaki
Quan Wu

Last updated by author(s): Dec 16th, 2021

Reporting Summary

Nature Research wishes to improve the reproducibility of the work that we publish. This form provides structure for consistency and transparency in reporting. For further information on Nature Research policies, see Authors & Referees and the Editorial Policy Checklist.

Statistics

For all statistical analyses, confirm that the following items are present in the figure legend, table legend, main text, or Methods section.

n/a	Confirmed	
☐	☒	The exact sample size (*n*) for each experimental group/condition, given as a discrete number and unit of measurement
☒	☐	A statement on whether measurements were taken from distinct samples or whether the same sample was measured repeatedly
☐	☒	The statistical test(s) used AND whether they are one- or two-sided<br>*Only common tests should be described solely by name; describe more complex techniques in the Methods section.*
☒	☐	A description of all covariates tested
☐	☒	A description of any assumptions or corrections, such as tests of normality and adjustment for multiple comparisons
☐	☒	A full description of the statistical parameters including central tendency (e.g. means) or other basic estimates (e.g. regression coefficient) AND variation (e.g. standard deviation) or associated estimates of uncertainty (e.g. confidence intervals)
☐	☒	For null hypothesis testing, the test statistic (e.g. *F*, *t*, *r*) with confidence intervals, effect sizes, degrees of freedom and *P* value noted<br>*Give P values as exact values whenever suitable.*
☒	☐	For Bayesian analysis, information on the choice of priors and Markov chain Monte Carlo settings
☒	☐	For hierarchical and complex designs, identification of the appropriate level for tests and full reporting of outcomes
☒	☐	Estimates of effect sizes (e.g. Cohen's *d*, Pearson's *r*), indicating how they were calculated

Our web collection on statistics for biologists contains articles on many of the points above.

Software and code

Policy information about availability of computer code

Data collection	There softwares were used for data collection:<br>Cell Sorter Software (version 2.1.5, Sony)<br>Olympus flouview version 4.1<br>Image reader LAS3000UVmini
Data analysis	These packages and softwares were used for data analysis:<br>WGCNA Cytoscape 3.7.2 Seurat 2.3.4 monocle 2.10.1 Trim Galore! 0.4.2 hisat2 2.1.0 Ribodiff 0.2.1 bowtie 1.2.1.1<br>Picard Samtools 1.5 MACS2 2.1.1 ngsplot 2.61 IGV 2.4.3 Deeptools 3.2.1 ChromHMM 1.14 DiffBind 2.10.0 CellProfiler 2.1.1<br>ViennaRNA 2.4.14 stringtie 1.3.6 TCC 1.22.1 ggplot2 3.3.5 pheatmap1.0.12 PMCMRplus 1.4.2 RColorBrewer 1.1-2 TCC (1.22.1)<br>Code for calculation birthdate and differentiation score was deposited at https://github.com/wuquan723/Birthdate-and-differentiation-score and DOI 10.5281/zenodo.5784971.

For manuscripts utilizing custom algorithms or software that are central to the research but not yet described in published literature, software must be made available to editors/reviewers. We strongly encourage code deposition in a community repository (e.g. GitHub). See the Nature Research guidelines for submitting code & software for further information.

Data

Policy information about availability of data

All manuscripts must include a data availability statement. This statement should provide the following information, where applicable:
- Accession codes, unique identifiers, or web links for publicly available datasets
- A list of figures that have associated raw data
- A description of any restrictions on data availability

All raw and proceeded data for scRNA, ChIP-seq and Ribo-seq generated in this study have been deposited in DNA Data Bank of Japan with accession number DRA009567, DRA009569 and DRA009729, DRA010791, DRA013203, and E-GEAD-348, E-GEAD-349 , E-GEAD-350 and E-GEAD-467 (https://ddbj.nig.ac.jp/resource/bioproject/PRJDB9278). Processed data generated in this study are also provided in the Supplementary Data files. We compared our scRNA data with previous data

GSE107122 (GEO Accession viewer (nih.gov)). To confirm the quality of our ChIP-seq data, we also compared our ChIP-seq H3K4me3 and H3K27me3 using E14 NSC with the published ChIP-seq data H3K4me3 (ENCSR172XOZ) and H3K27me3 (ENCSR831YAX) using E14 forebrain (https://www.encodeproject.org/).
Code for calculation birthdate and differentiation score was deposited at https://github.com/wuquan723/Birthdate-and-differentiation-score

Field-specific reporting

Please select the one below that is the best fit for your research. If you are not sure, read the appropriate sections before making your selection.

☒ Life sciences ☐ Behavioural & social sciences ☐ Ecological, evolutionary & environmental sciences

For a reference copy of the document with all sections, see nature.com/documents/nr-reporting-summary-flat.pdf

Life sciences study design

All studies must disclose on these points even when the disclosure is negative.

Sample size	We did not predetermine the sample size. Samples sizes were determined following best practices in the field.
Data exclusions	Small population (less than 10%) of Trp53 mutant mice showed defects in neural tube closure and caused exencephaly as early as E10 (Sah et al., 1995). These phenotype is not related with function of Fbl and these brains were not suitable for analysis of brain development. Therefore, mice showed exencephaly were excluded during dissection.
Replication	To analysis of mutant mice, we took at least three different sample for each genotypes at each stage. We repeated observed same phenotype. For scRNA, only one experiment was performed, but we performed bulk RNA-seq to confirm the result. For ChIP-seq, at least two independent experiment was performed following the guideline of encode ChIP data. All attempts at replication confirmed the reported findings.
Randomization	When we examined embryos from pregnant mice, litter mate embryos were analyzed in the random order.
Blinding	Because the mutant mice in this study can be easily distinguished during experiments, investigators were not blinded to sample identity during data collection and/or analysis.

Reporting for specific materials, systems and methods

We require information from authors about some types of materials, experimental systems and methods used in many studies. Here, indicate whether each material, system or method listed is relevant to your study. If you are not sure if a list item applies to your research, read the appropriate section before selecting a response.

Materials & experimental systems

n/a	Involved in the study	
☐	☒	Antibodies
☒	☐	Eukaryotic cell lines
☒	☐	Palaeontology
☐	☒	Animals and other organisms
☒	☐	Human research participants
☒	☐	Clinical data

Methods

n/a	Involved in the study	
☐	☒	ChIP-seq
☐	☒	Flow cytometry
☒	☐	MRI-based neuroimaging

Antibodies

Antibodies used

Fibrillarin (rabbit polyclonal, ab5821, abcam, Lot GR253838-1); Dilution rate: 1:1000; application: IHC.
Cleaved Caspase-3 (Asp175)(rabbit polyclonal, 9661S, Cell Signaling Technology, Lot-17); Dilution rate: 1:400; application: IHC.
Satb2 (mouse monoclonal, ab51502, abcam, Lot GR70015-12); Dilution rate: 1:400; application: IHC.
Tbr1 (rabbit polyclonal, ab31940, abcam, Lot GR204561-2); Dilution rate: 1:400; application: IHC.
Olig2 (goat polyclonal, AF2418, R&D System, Lot UPA0515071); Dilution rate: 1:400; application: IHC.
Pax6 (rabbit polyclonal, PRB-278P, Covance, Lot B214847); Dilution rate: 1:400; application: IHC.
Sox2 (goat polyclonal, sc-17320, Santa Cruz, Lot E2912); Dilution rate: 1:400; application: IHC.
Tbr2/EOMES (rat monoclonal, clone Dan11mag; eBioscience at Thermo Fisher, Lot 4348412); Dilution rate: 1:400; application: IHC.
Phospho-Histone H3 (Ser10) (rabbit polyclonal, 06-570, Millipore, Lot DAM1416518); Dilution rate: 1:400; application: IHC.
Brn-2 (goat polyclonal, sc-6029, Santa Cruz, Lot A1014); Dilution rate: 1:400; application: IHC.
FoxP2 (goat polyclonal, sc-21069, Santa Cruz, Lot G2911); Dilution rate: 1:400; application: IHC.
GFP (chick polyclonal, GFP-1020, aves, GFP697986 or GFP879484); Dilution rate: 1:400; application: IHC.
Dmrt3 (rabbit polyclonal, a gift from D. Konno); Dilution rate: 1:1000; application: IHC.
Alexa Flour 488, cy3, or 647; Jackson ImmunoResearch; Dilution rate: 1:1000; application: IHC.
Ezh2 (mouse monoclonal, 5246S, Cell Signaling Technology, Lot 9); Dilution rate: 1:1000; application: western blot.

Histone H3 (rabbit monoclonal, 4499, Cell Signaling Technology, Lot 1); Dilution rate: 1:1000; application: western blot.
Kdm6b (rabbit polyclonal, NBP1-06640, Novus Biologicals, Lot F-4); Dilution rate: 1:1000; application: western blot.
Sox2 for western blotting (rabbit polyclonal, ab75179, Abcam, Lot 626574); Dilution rate: 1:1000; application: western blot.
alpha-Tubulin (mouse monoclonal, clone DM1A, T9026, Sigma-Aldrich, Lot 081M4861); Dilution rate: 1:1000
H3K27me3 (rabbit monoclonal,Cell Signaling Technology, #9733, Lot 8)
H3K4m3 (mouse monocle, wako, 307-34813, Lot 14004)

Validation

Commercial antibodies were validated as described on website of each company. Dmrt3 antibody was validated as describe in Konno et al., 2012.
GFP: species; mouse, application; IHC, relevant citation; Tsunekawa et al., Development 143, 3216-3222 (2016)
Pax6: species; mouse, application; IHC, relevant citation; Konno et al., Nat. Cell Biol. 10, 93–101 (2008), Fietz et al., Nat. Neurosci. 13, 690-699 (2010)
Sox2: species; mouse, application; IHC, relevant citation; Konno et al., Development 146, dev174243 (2019)
Tbr2/EOMES: species; mouse, application; IHC, relevant citation; Tsunekawa et al., Development 143, 3216-3222 (2016), Konno et al.,Development 146, dev174243 (2019)
phospho-histone H3 (Ser10, rabbit polyclonal): species; mouse, application; IHC, relevant citation; Kawaue et al., Nat. Commun., 10, 2780
Fibrillarin (rabbit polyclonal, ab5821, abcam, Lot GR253838-1); Dilution rate: 1:1000; application: IHC. https://www.abcam.com/fibrillarin-antibody-nucleolar-marker-ab5821.html
Cleaved Caspase-3 (Asp175)(rabbit polyclonal, 9661S, Cell Signaling Technology, Lot-17); application: IHC.https://www.cellsignal.jp/products/primary-antibodies/cleaved-caspase-3-asp175-antibody/9661
Satb2 (mouse monoclonal, ab51502, abcam, Lot GR70015-12);application: IHC.https://www.abcam.co.jp/satb2-antibody-satba4b10-c-terminal-ab51502.html
Tbr1 (rabbit polyclonal, ab31940, abcam, Lot GR204561-2); application: IHC. Konno et al.,Development 146, dev174243 (2019)
Olig2 (goat polyclonal, AF2418, R&D System, Lot UPA0515071); application: IHC. https://www.rndsystems.com/products/human-mouse-rat-olig2-antibody_af2418
Brn-2 (goat polyclonal, sc-6029, Santa Cruz, Lot A1014); application: IHC. Konno et al.,Development 146, dev174243 (2019)
FoxP2 (goat polyclonal, sc-21069, Santa Cruz, Lot G2911); application: IHC. https://www.scbt.com/p/foxp2-antibody-n-16
Alexa Flour 488, cy3, or 647; Jackson ImmunoResearch; application: IHC. https://www.jacksonimmuno.com/technical/products/conjugate-selection/alexa-fluor
Ezh2 (mouse monoclonal, 5246S, Cell Signaling Technology, Lot 9); application: western blot. https://www.cellsignal.com/products/primary-antibodies/ezh2-d2c9-xp-rabbit-mab/5246
Histone H3 (rabbit monoclonal, 4499, Cell Signaling Technology, Lot 1); application: western blot.https://www.cellsignal.com/products/primary-antibodies/histone-h3-d1h2-xp-rabbit-mab/4499
Kdm6b (rabbit polyclonal, NBP1-06640, Novus Biologicals, Lot F-4); application: western blot. https://www.novusbio.com/products/lysine-k-specific-demethylase-6b-kdm6b-jmjd3-antibody_nbp1-06640
H3K27me3 (rabbit monoclonal, Cell Signaling Technology, #9733); application: ChIP-seq; https://www.cellsignal.jp/products/primary-antibodies/tri-methyl-histone-h3-lys27-c36b11-rabbit-mab/9733
H3K4me3 (monocle, Wako, 307-34813); application: ChIP-seq; https://labchem-wako.fujifilm.com/jp/product/detail/W01M1630-3481.html

Animals and other organisms

Policy information about studies involving animals; ARRIVE guidelines recommended for reporting animal research

Laboratory animals

Fbl and Trp53 mutant mice was maintained in C57BL/6 background. The reporter mouse line: pHes1–d2EGFP was maintained in ICR background. Wild type mice used for inhibitors treatment and IRES activity tests were maintained in ICR background. All female mice used in this study are older than 2 months. We did not distinguish the sex of embryos.

Wild animals

No wild animals were used in the study.

Field-collected samples

No field collected samples were used in the study.

Ethics oversight

All animal procedures were performed in accordance with the guidelines for animal experiments at RIKEN Center for Biosystems Dynamics Research.

Note that full information on the approval of the study protocol must also be provided in the manuscript.

ChIP-seq

Data deposition

☒ Confirm that both raw and final processed data have been deposited in a public database such as GEO.

☒ Confirm that you have deposited or provided access to graph files (e.g. BED files) for the called peaks.

Data access links
May remain private before publication.

All raw data for ChIP-seq are available at the DNA Data Bank of Japan with accession number DRA009567.
Proceeded data can be reviewed at https://ddbj.nig.ac.jp/gea/reviewer/login
accession number: E-GEAD-349;

Files in database submission

170909_E11_H3K4me3_bowtie_md_peaks.txt
170909_E11_H3K27me3_bowtie_md_peaks.txt
170909_E12_H3K4me3_bowtie_md_peaks.txt

170909_E12_H3K27me3_bowtie_md_peaks.txt
171003_E14_H3K4me3_bowtie_md_peaks.txt
171003_E14_H3K27me3_bowtie_md_peaks.txt
171003_E14_H3K27me3D_bowtie_md_peaks.txt
E11_H3K4me3_bowtie_md_peaks.txt
E11_H3K27me3_bowtie_md_peaks.txt
E12_H3K4me3_bowtie_md_peaks.txt
E12_H3K4me3D_bowtie_md_peaks.txt
E12_H3K27me3_bowtie_md_peaks.txt
E14_H3K4me3_bowtie_md_peaks.txt
E14_H3K27me3_bowtie_md_peaks.txt
181102_E14_Con7_H3K27me3_sort_md_peaks.txt
181107_E14_Het9_H3K27me3_sort_md_peaks.txt
181109_E14_Het9_H3K4me3_sort_md_peaks.txt
190209_E14_Con11_H3K27me3_sort_md_peaks.txt
190209_E14_Con12_H3K27me3_sort_md_peaks.txt
190209_E14_DKO1_H3K27me3_sort_md_peaks.txt
190209_E14_DKO3_H3K27me3_sort_md_peaks.txt
190209_E14_Het9_H3K27me3_sort_md_peaks.txt
190215_E14_Con11_H3K4me3_sort_md_peaks.txt
190215_E14_Con12_H3K4me3_sort_md_peaks.txt
190215_E14_DKO1_H3K4me3_sort_md_peaks.txt
190215_E14_DKO3_H3K4me3_sort_md_peaks.txt
190215_E14_Het9_H3K4me3_sort_md_peaks.txt

Genome browser session
(e.g. UCSC)

N/A

Methodology

Replicates

E11_H3K4me3: n=2
E12_H3K4me3: n=3
E14_H3K4me3: n=2
E11_H3K27me3: n=2
E12_H3K27me3: n=2
E14_H3K27me3: n=3
E14 Fbl+/+ _H3K4me3: n=2
E14 FblΔ/+ _H3K4me3: n=2
E14 DKO _H3K4me3: n=2
E14 Fbl+/+ _H3K27me3:n=3
E14 FblΔ/+ _H3K27me3:n=2
E14 DKO _H3K27me3:n=2

Sequencing depth

Basically, H3K4me3 and input samples were read at around 20M/sample and H3K27me3 samples were read at around 40M/sample.
Sample #Total Reads after Triming #Reads with at least one reported alignment
170315_E11_Input 19693015 14232782 (72.27%)
170909_E11_H3K4me3 19037436 14907936 (78.31%)
170909_E11_H3K27me3 34538599 24518181 (70.99%)
170909_E11_Input 19150382 13820499 (72.17%)
170909_E12_H3K4me3 18912609 13694482 (72.41%)
170909_E12_H3K27me3 37968650 27580995 (72.64%)
170909_E12_Input 19449821 13915969 (71.55%)
170909_E14_Input 16885298 11754640 (69.61%)
171003_E14_H3K4me3 18023510 14945570 (82.92%)
171003_E14_H3K27me3 33725613 24995076 (74.11%)
171003_E14_H3K27me3D 33815003 24201819 (71.57%)
E11_H3K4me3 18686861 13255546 (70.94%)
E11_H3K27me3 34727030 23895204 (68.81%)
E12_H3K4me3 18289879 13375907 (73.13%)
E12_H3K4me3D 19980178 14321880 (71.68%)
E12_H3K27me3 36003392 25571035 (71.02%)
E12.5_Input 17591766 12837489 (72.97%)
E14_H3K4me3 18676150 13396289 (71.73%)
E14_H3K27me3 36687679 26249687 (71.55%)
E14_Input 17773228 12622971 (71.02%)
181102_E14_Con7_H3K27me3 39061865 28324116 (72.51%)
181107_E14_DKO1_H3K4me3 30471680 22578053 (74.10%)
181107_E14_Het9_H3K27me3_ 46479122 33697399 (72.50%)
181107_E14_Input 45689822 33808078 (73.99%)
181109_E14_DKO1_H3K27me3 43230631 31428846 (72.70%)
181109_E14_Het9_H3K4me3 37431018 27758156 (74.16%)
190209_E14_Con11_H3K27me3 40991646 31562997 (77.00%)
190209_E14_Con11_Input 20757813 15171707 (73.09%)
190209_E14_Con12_H3K27me3 42002510 31988986 (76.16%)

190209_E14_Con12_Input 20272495 14899539 (73.50%)
190209_E14_DKO1_H3K27me3 41060986 30970961 (75.43%)
190209_E14_DKO1_Input 18479146 13576262 (73.47%)
190209_E14_DKO3_H3K27me3 44782558 34584417 (77.23%)
190209_E14_DKO3_Input 18914131 14125992 (74.68%)
190209_E14_Het9_H3K27me3 43745291 32850010 (75.09%)
190209_E14_Het9_Input 20348434 14623544 (71.87%)
190215_E14_Con11_H3K4me3 19559521 14843078 (75.89%)
190215_E14_Con12_H3K4me3 20600834 15624972 (75.85%)
190215_E14_DKO1_H3K4me3 22402865 17674072 (78.89%)
190215_E14_DKO3_H3K4me3 21092997 16086368 (76.26%)
190215_E14_Het9_H3K4me3 19734650 14866333 (75.33%)

Antibodies

H3K27me3 (rabbit monoclonal,Cell Signaling Technology, #9733, Lot 8)
H3K4m3 (mouse monocle, wako, 307-34813, Lot 14004)

Peak calling parameters

Peaks of ChIP-seq were called using MACS2 (2.1.1). Q-value to cutoff H3K4me3 peaks was set at 0.01. For call peaks of H3K27me3 --broad function was used and q-value was set at 0.01 and 0.05 to cutoff narrow/strong regions or broad/weak regions, respectively.

Data quality

We observed high correlation between each duplicated sample and between our samples with published Encode ChIP-seq data from whole brains.

Software

Sequenced reads were quality controlled and trimmed with Trim Galore! (https://www.bioinformatics.babraham.ac.uk/projects/trim_galore/). Reads were then mapped onto mouse genome mm10 using bowtie with the parameter –m1 --best --strata. Mapped sam file were transferred into bam file and sorted with samtools. Duplicates were marked and removed with Picard. Peaks of ChIP-seq were called using MACS2 (2.1.1) with parameter. For each sample, specifying input were used as control.
Deeptools was used to calculation correlation of each data set. The alignment files were binned using multiBamSummary function with default setting and pearson correlation was calculated using plotCorrelation function. To confirm the quality of our ChIP data, we also compared our H3K4me3 and H3K27me3 ChIP-seq using E14.5 neural stem cells with published H3K4me3 (ENCSR172XOZ) and H3K27me3 (ENCSR831YAX) ChIP-seq data using E14.5 forebrain (https://www.encodeproject.org/).
ChromHMM was used to evaluate state transition between different stages. The alignment files of H3K4me3 and H3K27me3 in each stage were binned into 200-bp bins using BinarizeBam. Then, we established the model with 4 emission states (H3K4me3-only, H3K27me3-only, bivalent and none) and trained with binned data using LearnModel command. These segmentation files with state information was used to plot alluvial plotting.
DiffBind package in R was used to find differential peak intensity between samples and different stages and to visualize data with PCA plotting. To do so, overlapping peaks among each samples were isolated and calculate reads on these consensus peaks using dba.count function. As a result, the binding affinity matrix in which each column indicates consensus peaks and each row indicates normalized reads counting can be produced. The matrix was used to plotting PCA using ggplot function in R. The differential binding affinity was calculated using dba.contrast and dba.analyze function. MA plotting was draw by dba.plotMA function.

Flow Cytometry

Plots

Confirm that:

☒ The axis labels state the marker and fluorochrome used (e.g. CD4-FITC).

☒ The axis scales are clearly visible. Include numbers along axes only for bottom left plot of group (a 'group' is an analysis of identical markers).

☒ All plots are contour plots with outliers or pseudocolor plots.

☒ A numerical value for number of cells or percentage (with statistics) is provided.

Methodology

Sample preparation

To isolated Hes1 positive neural stem cells (NSCs), dorsal cortices were dissected from Hes1-d2-EGFPTg/+ mice. Cortices were dissociated with 0.05% trypsin with Hanks'Balanced Salt Solution (HBSS) (–) at 37 °C for 10 min. After centrifugation at 1000g for 5 min, cells were re-suspended with 0.375% BSA/HBSS(-) by gentle pipetting 15 to 20 times. Re-suspended cells were filtered with 35 µm filter (Falcon) and sorted into sorting buffer (20 ng/ml human basic FGF (Peprotech), 1XB27 RA- (Gibco), in Dulbecco's Modified Eagle Medium (DMEM) F12+GlutaMax (Gibco)) by a cell sorter (SH800, SONY) equipped with 130 µm sorting chips (SONY, LE-C3113).

Instrument

Sony SH800

Software

Cell sorter software (v2.1.5 sony)

Cell population abundance

Cell population in cell cycle analysis was provide in the source date Figure 4.

Gating strategy

FSC-A and BSC-A was used to gate single cell population. For EGFP sorting, wild type cells without fluorescence were used negative control and the gate was set to exclude these negative cells. Compensation was performed before sorting EGFP and BFP double positive cells and gate was set to exclude these negative control. For cell cycle analysis, gate to set around the peak of each cell cycle state.

☒ Tick this box to confirm that a figure exemplifying the gating strategy is provided in the Supplementary Information.
