# Supplementary material for: Selective translation of epigenetic modifiers affects the temporal pattern and differentiation of neural stem cells
Source: Nat Commun. 2022 Jan 25;13:470. doi: 10.1038/s41467-022-28097-y (PMC8789897; doi:10.1038/s41467-022-28097-y)
Supplement: Supplementary file 6 — Source Data [file 41467_2022_28097_MOESM6_ESM.zip › Source_Date_2021/Western_Blotting.pdf]

Fig. 1g

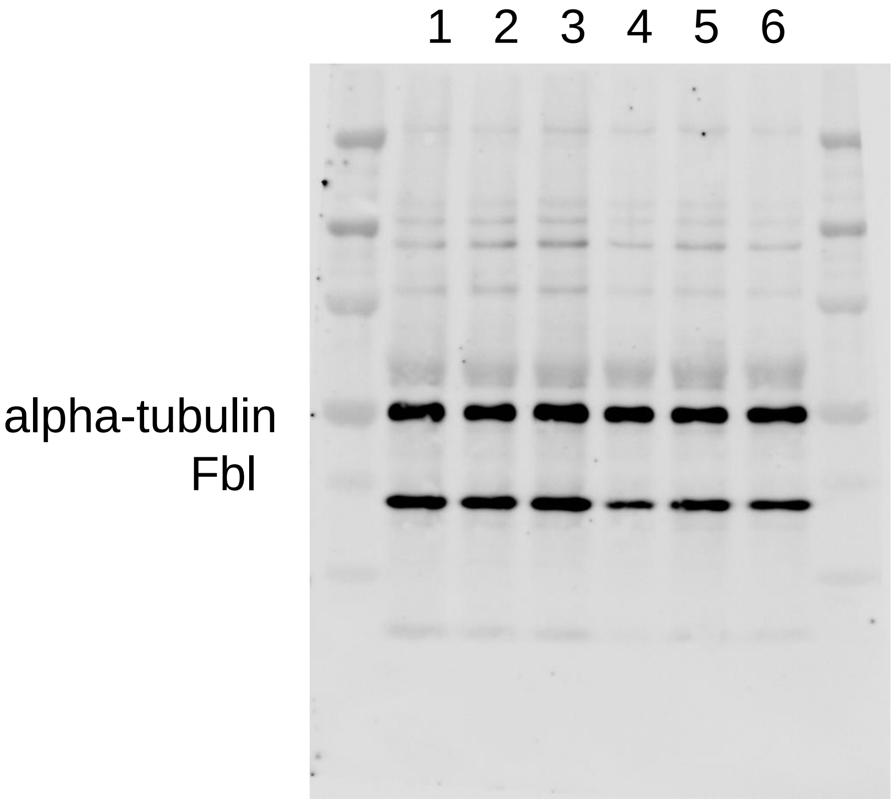

1-3. E11 Hes1+ NSC

4-6. E14 Hes1+ NSC

Fig. 5d

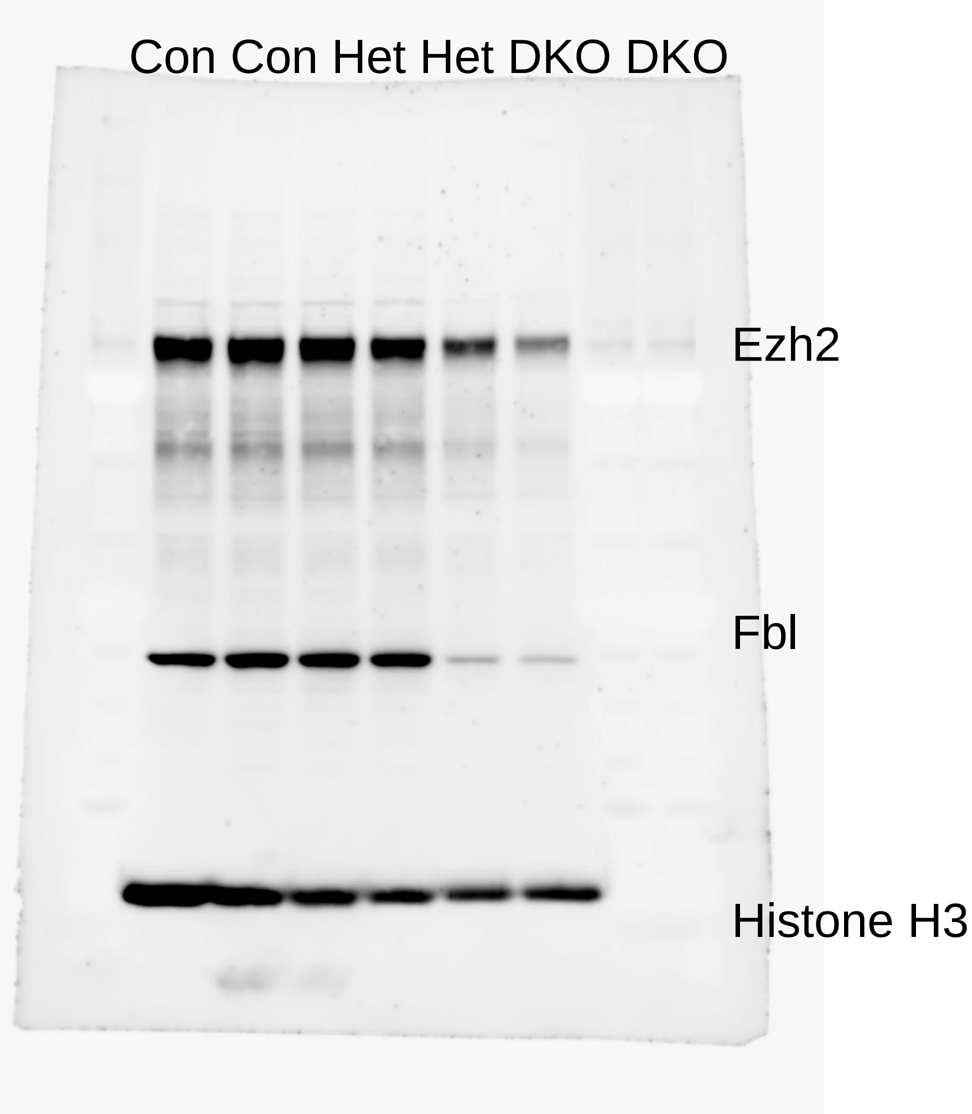

Fig. 5d

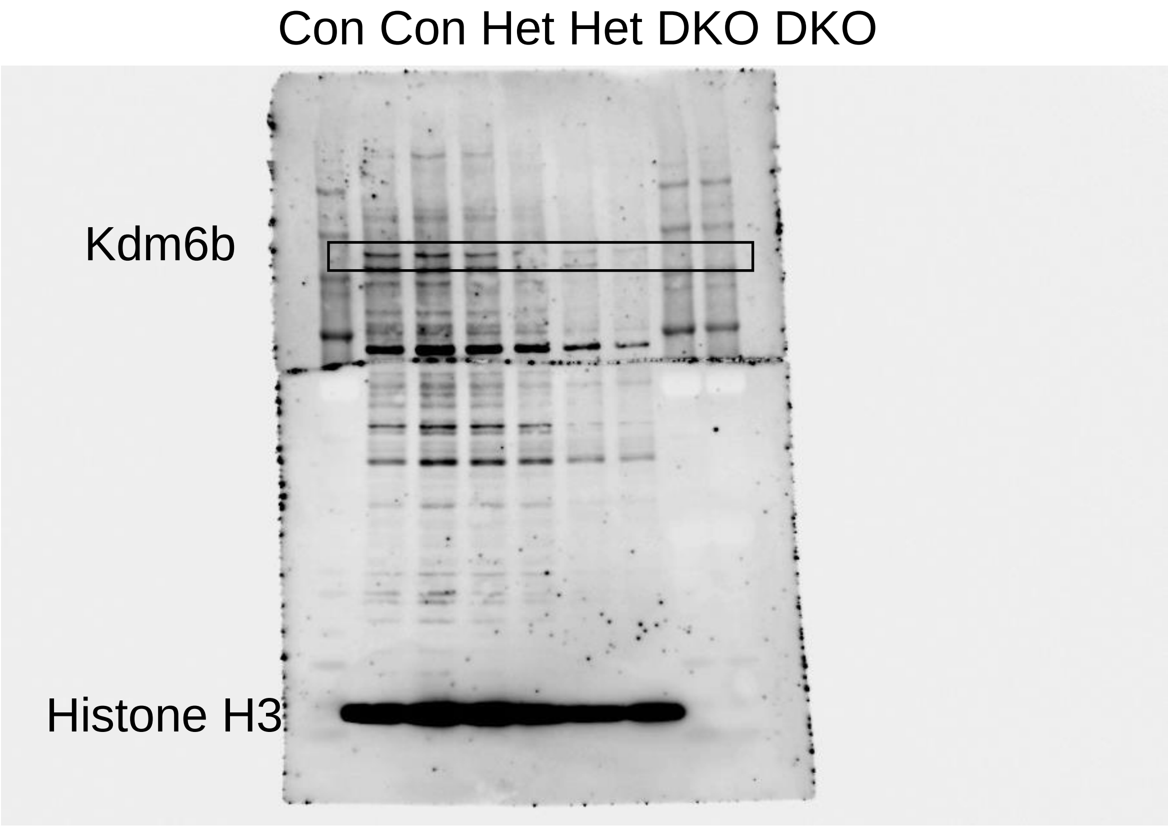

Fig. 5d

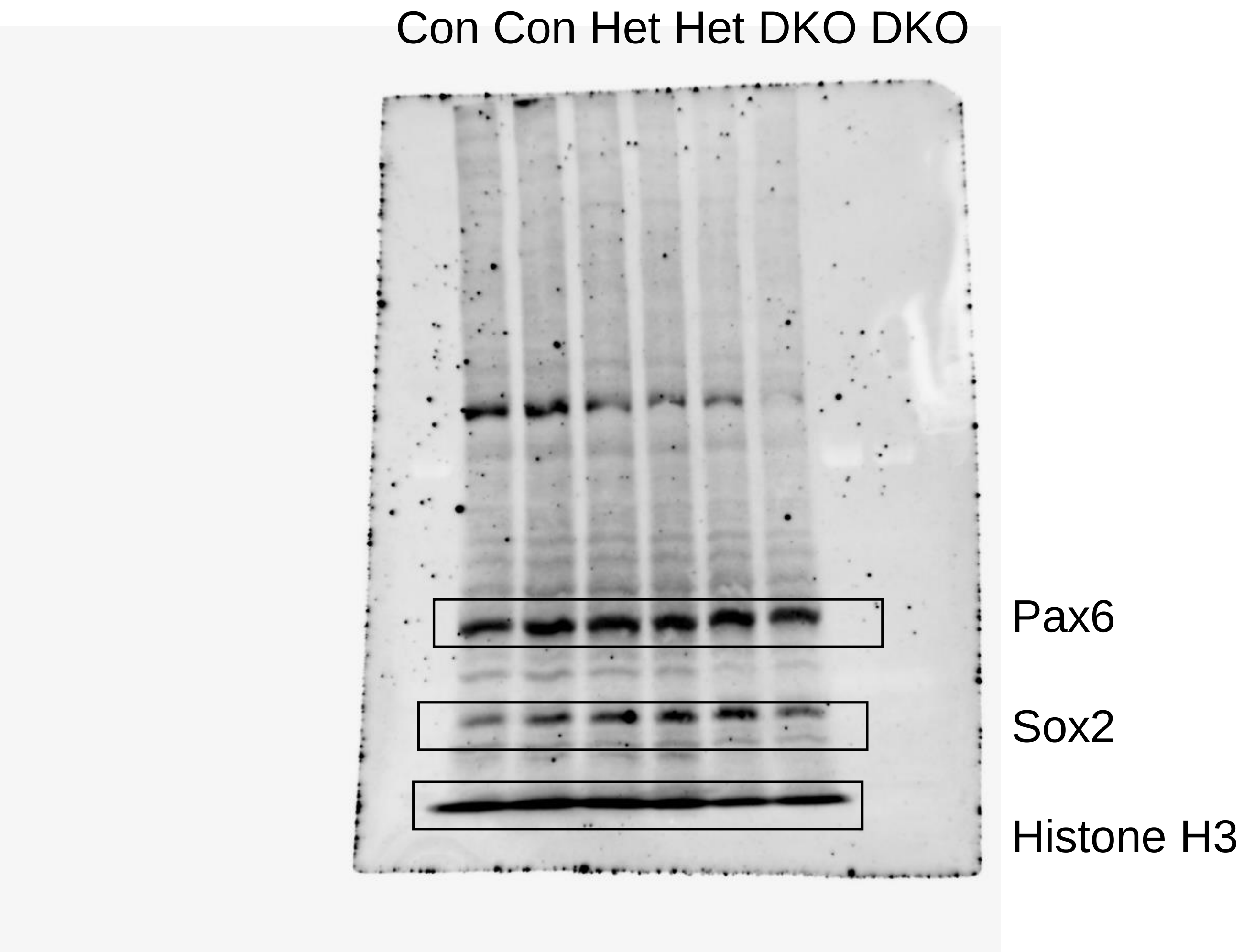

Supplementary Fig. 7i

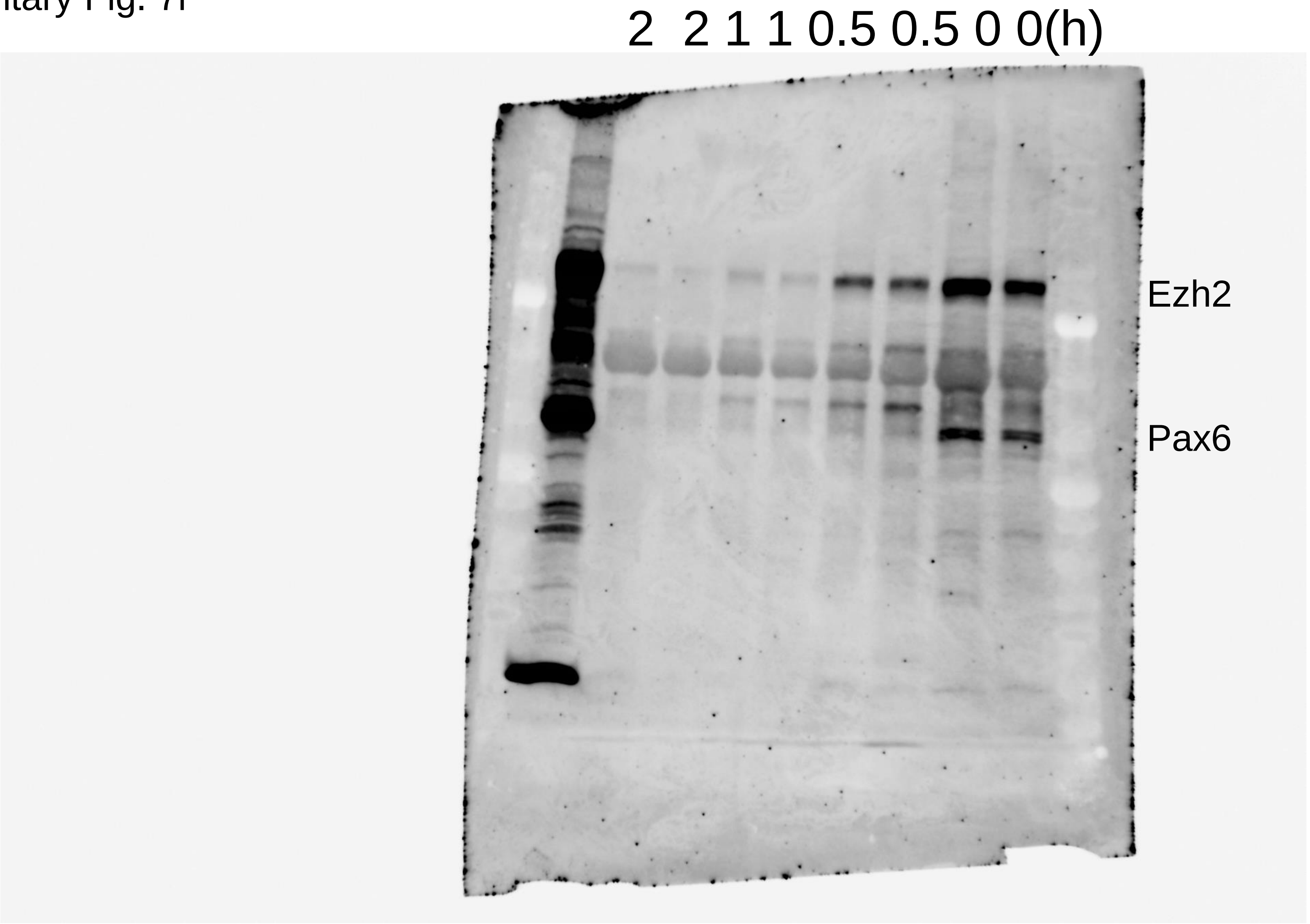

Supplementary Fig. 10a

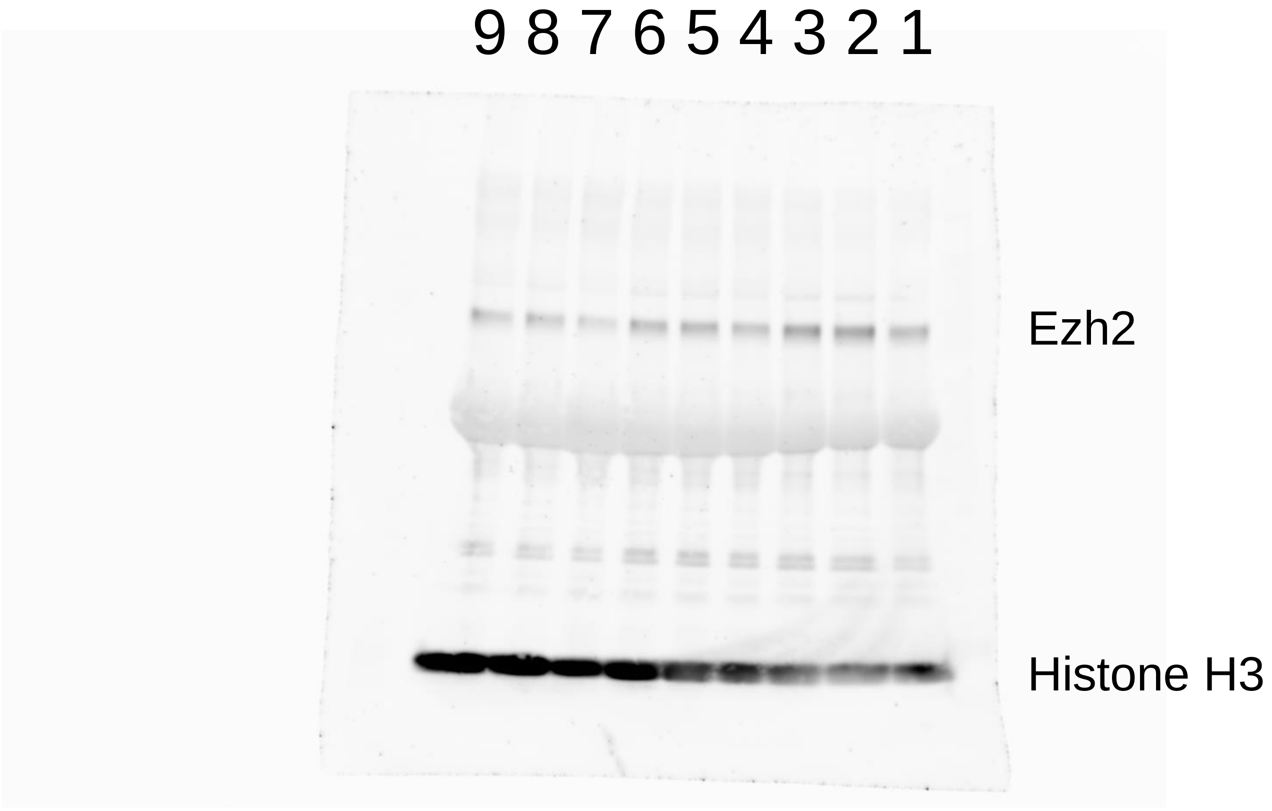

1. Con1
2. Con2
3. Con3
4. gRNA1_1
5. gRNA1_2
6. gRNA1_3
7. gRNA2_1
8. gRNA2_2
9. gRNA2_3

Supplementary Fig. 10b

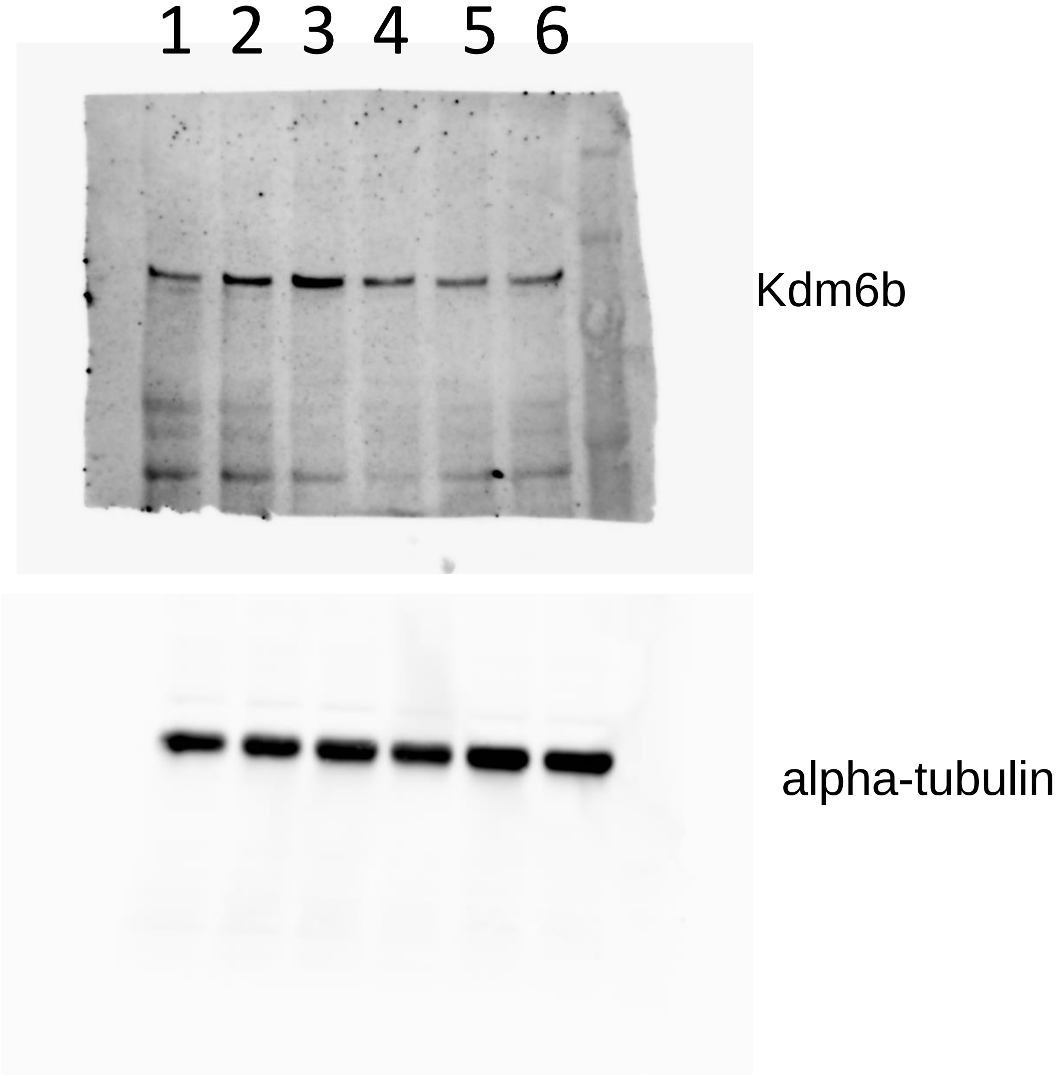

1. Con1
2. Con2
3. Con3
4. gRNA_1
5. gRNA_2
6. gRNA_3
